# Fairness Norms and Theory of Mind in an Ultimatum Game: Judgments, Offers, and Decisions in School-Aged Children

**DOI:** 10.1371/journal.pone.0105024

**Published:** 2014-08-13

**Authors:** Ilaria Castelli, Davide Massaro, Cristina Bicchieri, Alex Chavez, Antonella Marchetti

**Affiliations:** 1 Research Unit on Theory of Mind, Department of Psychology, Università Cattolica del Sacro Cuore, Milano, Italy; 2 Behavioral Ethics Lab, Departments of Philosophy and Psychology, University of Pennsylvania, Philadelphia, Pennsylvania, United States of America; 3 Department of Psychology, University of Michigan, Ann Arbor, Michigan, United States of America; Middlesex University London, United Kingdom

## Abstract

The sensitivity to fairness undergoes relevant changes across development. Whether such changes depend on primary inequity aversion or on sensitivity to a social norm of fairness is still debated. Using a modified version of the Ultimatum Game that creates informational asymmetries between Proposer and Responder, a previous study showed that both perceptions of fairness and fair behavior depend upon normative expectations, i.e., beliefs about what others expect one should do in a specific situation. Individuals tend to comply with the norm when risking sanctions, but disregard the norm when violations are undetectable. Using the same methodology with children aged 8–10 years, the present study shows that children's beliefs and behaviors differ from what is observed in adults. Playing as Proposers, children show a self-serving bias only when there is a clear informational asymmetry. Playing as Responders, they show a remarkable discrepancy between their normative judgment about fair procedures (a coin toss to determine the offer) and their behavior (rejection of an unfair offer derived from the coin toss), supporting the existence of an outcome bias effect. Finally, our results reveal no influence of theory of mind on children's decision-making behavior.

## Introduction

Recent research on decision-making in children has shown strong inequity aversion in Ultimatum games [1]. In such games, children consistently reject unfair offers [2]. This might be due to primary inequity aversion or to sensitivity to social norms of fairness. The interpretation of such results would differ depending on the reason why offers are rejected. Inequity aversion motives would be independent of the procedure that generated the offer, whereas rejection due to social norms would be sensitive to process and intentions. For example, adults who receive a low offer from a random device (coin toss, dice, roulette, etc.) normally accept the offer, yet almost 50% reject offers of 20% or less coming from an individual [3]. According to the view of social norms we adopt here [4], the decision to accept or reject an offer depends upon the empirical and normative expectations entertained by the individual. We adopt here Bicchieri's “constructivist” definition of social norm [4]: A norm exists if a sufficient number of people believe (a) that it exists and applies to a specific class of situations, (b) that most people are following it in those situations and (c) that most people believe one ought to follow it. Individuals will prefer to follow the norm whenever all these conditions are satisfied. An *empirical expectation* is the expectation that other people in the relevant population comply with the norm, and a *normative expectation* is the belief that other people in the relevant population expect the individual to obey the norm and may sanction transgressions. Individuals will thus have a *conditional* preference to follow a fairness norm depending upon the existence of the relevant empirical and normative expectations [4]. It is also important to observe that – in order to assess the presence of a norm – we have to independently measure individuals' normative expectations. That is, whenever there is a general agreement that most members of the relevant group consider certain behaviors to be fair, we can be reasonably sure that a shared norm of fairness exists.

Even if we are sure a fairness norm applies to a given situation, consensus does not imply universal conformity. Bicchieri and Chavez [5] manipulated adults' expectations about fairness by creating informational asymmetries about the offer choices available to the Proposer in an Ultimatum game, and found that behavior varies accordingly. Proposers and Responders did show a remarkable degree of agreement in their beliefs about which choices are considered fair by a majority of participants, and it is precisely this mutual consistency in normative second-order beliefs (normative expectations) that is a mark of the existence of a shared norm. Moreover, when normative expectations are present without the sanctioning element, so that such expectations can be violated at no cost, Bicchieri and Chavez show that individuals tend to disobey the norm, since the victim will not be able to distinguish an intentional action from chance [6]. The results of the Bicchieri and Chavez experiment show that most (adult) participants are sensitive to the manipulation of information. Furthermore, for this manipulation to be understood and exploited, participants must have an advanced meta-representational ability. For example, a subject must be able to replicate others' beliefs as well as others' potential responses to a range of actions. Meta-representations are at the core of theory of mind, the ability to predict and interpret our own and others' behavior in terms of mental states [7]. The acquisition of the elementary level of this competence appears around four years of age, when children solve the first-order false belief task that shows the ability of first-level recursive thinking (I think that you think…) [8]. This ability evolves when the child, approximately at the age of eight, solves the second-order false belief task, showing the ability to use second-level recursive thought (“I think that you think that she/he thinks”…) [9]. The theory of mind has been called the “ability among abilities”, as it is not simply an independent competence used in specific and restricted contexts (for example, the false belief task), but it is one of the most important cognitive skills used in social and strategic reasoning [10]–[12]. This fact is particularly evident in studies that explore theory of mind development in a life-span perspective [13] and in age-related clinical conditions [14], [15].

Experiments with the Ultimatum game and related games can help us gain insight in what people believe to be fair outcomes in cases where some good is to be shared among claimants. In such games, a Proposer must offer a share of some good (usually money) provided by the experimenter to an anonymous Responder, who can accept or reject the offer. If the offer is rejected, both players get nothing. Such games offer the opportunity to combine strategic thinking with sensitivity to fairness in various degrees. Sensitivity to fairness may mean two very different things. On the one hand, it may refer to a basic aversion to unequal outcomes. On the other hand, it may refer to awareness of a social norm of fairness, and strategic application of the norm in all cases in which transgressing it leads to negative consequences. Experiments on Ultimatum games usually do not make this distinction that is nonetheless crucial in predicting future behavior. Inequity aversion should be a stable disposition, whereas norm-following will predictably depend on players' expectations. Experimental results support the second hypothesis [5], since manipulating expectations led players to radically change offer behavior.

Ultimatum games experiments played by children do not usually distinguish between the two interpretations of ‘sensitivity to fairness’. They do show important age differences in children's behavior in the Ultimatum Game [1], [16]–[18] as well as in the link between fair behavior and theory of mind [2], [19]–[21]. Sally and Hill [20], for example, found that children's progressive mentalizing ability explains greater avoidance of unsuccessful ultimatum proposals in older children. They explicitly refer to fairness norms, and state that “the development of Theory-of-Mind skills may help the child first to recognize and act upon relevant norms of behavior, such as fairness, and later, to stretch those norms and improvise away from them when the situation calls for it” (p. 94).

The present research aimed to replicate the Bicchieri and Chavez [5] experiment with school-age children. We were interested in assessing whether children are sensitive to fairness (either as inequity aversion or a norm of fairness) and whether informational manipulations lead to behavioral changes. In the latter case, responsiveness to information manipulations would suggest that children prefer to follow a fairness norm on condition of having certain expectations, but not unconditionally. On the other hand, unresponsiveness and a preference for fair outcomes would indicate a more basic disposition toward fairness, independent of the existence of norms that dictate fair behavior. Our results are mixed. When in the Proposer's role, children are indeed responsive to information manipulations. However, Responders uniformly rejected unfair offers in any condition. Children, as opposed to adults, overweight consequences to the detriment of procedures that they otherwise find fair. We also wanted to assess the children's theory of mind, operationalized as false belief understanding, and relate different levels of false belief understanding to fair behaviors. We used the false belief task that is considered the litmus test of theory of mind, even if it is not exhaustive of the full mentalizing abilities of children [10], [22].

The four important results we draw are that: a) there is convergence of children's normative expectations about what the majority believes is fair. We can therefore conclude that children share and are aware of fairness norms. However, differently from adults, both an equal split and the use of a random device (a coin toss) are considered equally fair by almost all participants. b) Children are sensitive to information manipulations. In different information conditions, children make different offers. In other words, it appears that children decide how to split the good on the basis of what they think that the other children know about the set of available offers. c) Unfair offers, however obtained, are uniformly rejected in all conditions. In particular, there is an inconsistency between the acknowledgment of the procedural fairness of a coin toss and acceptance of outcomes that result from it. Although the use of a coin toss is universally perceived as fair by children, an unfavorable result is consistently rejected. This result seems to suggest that inequity aversion is a more primitive disposition than the ability to recognize the causal link between the procedure (coin toss) – chosen because considered fair - and the outcome of that procedure. d) Measures of first and second order false belief understanding do not seem to influence decision making. Although the idea that theory of mind is involved in decision making remains theoretically grounded, the tasks used to measure theory of mind may not have grasped aspects of this skill involved in the ultimatum game employed in the current research.

## Methods

### Participants

Participants of the study described in the paper have been treated according to the APA ethical standards and informed consent was obtained. The study was approved by the Local Ethic Committee (Università Cattolica del Sacro Cuore, Milan, Italy). Official authorizations to carry on the research were provided by the Director of the School and by the teachers of the classes involved. Informed written consent was obtained from the parents of each participant. One hundred and two children from a primary school (middle SES) in northern Italy took part in the research. They were divided in two age groups: young (N = 42, male = 24, female = 18, mean age = 8.8 years old) and old (N = 60, male = 36, female = 24, mean age = 10.9 years old).

### Game Paradigm

The experimental design was the same as the one devised by Bicchieri and Chavez [5] with a variant of the Ultimatum Game – UG – [23]. Playing with children, we did not use money, but tokens that would have been changed into candies or stickers according to the child's preference. Before explaining the rules of the game, the child was asked to state her/his preference between candies and stickers, and was told that he/she would have played a game where he/she could win a number of them. Then the child was explained the rules of the game: one participant, the Proposer, received 10 tokens – provided by the experimenter – and then proposed a division of those tokens to a Responder. If the Responder accepted, both players received the amounts specified in the proposal. If the Responder rejected, both players received nothing. Following the variant by Bicchieri and Chavez [5], the Proposer chose one from the following three options:

(5,5) - to propose 5 tokens for the Proposer, and 5 tokens for the Responder;

(8,2) - to propose 8 tokens for the Proposer, and 2 tokens for the Responder; and

Coin - to let the outcome of a fair coin flip determine the proposal: heads corresponded to (5,5), and tails to (8,2).

### Procedure

Children were introduced to the experimenter, who explained that they were going to play some games in two quiet rooms at school. The children were randomized into one or two school rooms upon their arrival, which determined whether they would be a Proposer or a Responder for the duration of the study. Once in the room with the experimenter, children received the instructions that explained the UG, that they would play three such games with a different child chosen at random in the other room, that all choices and responses were strictly anonymous, and that children would be paid with candies or stickers (as they would prefer) at the end of the experimental session. To be sure that children understood the anonymity condition, each child was given an envelope containing some tickets. Each ticket had a symbol on it, i.e. a cross, a dot, a star and so on. The child was asked to secretly pick up one ticket, and was told that the particular symbol on the ticket would be his/her only identifier. Before each game the experimenter provided additional instructions to participants. Finally, Proposers completed proposal forms and Responders responded to them. The Supporting Information document provides the full set of instructions (see Document S1), the proposal forms (see Document S2) and the questionnaires (see Document S3 and Document S4) that were used to measure Responders' first-order normative beliefs and Proposers' and Responders' second-order normative beliefs (expectations). These measured belief variables allowed us to assess the presence of norms, and to determine which beliefs were most relevant to Proposers' choices.

### Salience

In each information condition, prior to making their choices, all Responders completed a questionnaire that measured their first- and second-order normative beliefs (see Documents S3 and S4). The questionnaire asked whether the Responder found each of the available choice options fair, and also what they thought the majority of other Responders found fair. The questionnaire was aimed at assessing whether there was an agreement in Responders' normative expectations, an indicator of (as well as a necessary condition for) the existence of a social norm. In addition, half of the experimental sessions included an incentive-based questionnaire for Proposers, which they completed in each information condition (See Document S4). These questionnaires were designed to 1) make fairness norms more salient, and 2) test for an agreement between Responders' normative expectations and Proposers' beliefs about them. The other half of the sessions just included the Responders' questionnaires.

### Information Conditions

Participants played three Ultimatum Games under different information conditions in a fixed-order, within-subjects design.

1. In the full information condition, Proposers marked on a proposal form whether their choice was (5,5), (8,2), or Coin. Subsequently, the experimenter in the room of Responders flipped a coin. On any forms on which the Proposer chose Coin, the experimenter marked (5,5) or (8,2), based on the coin flip outcome. Thus, all participants understood that the Coin option was available and that Responders would know if the Proposer with whom they were paired chose Coin.

2. In the private information condition, Responders did not know that Coin was available to Proposers, and Proposers were aware of this fact. To create this informational asymmetry, we eliminated Coin from the proposal form, but allowed Proposers to choose Coin by leaving the remaining options ((5,5) and (8,2)) unmarked on the form. An experimenter in the Proposers' room then flipped a coin. On any forms on which the Proposer chose Coin, the experimenter marked (5,5) or (8,2), based on the coin flip outcome. Thus, Responders only saw a form with either (5,5) or (8,2) marked, and were unaware of the existence of the Coin option.

3. In the limited information condition, all participants knew that the Coin option was available, but that the Responder would not be able to distinguish whether the Proposer chose (5,5) or (8,2) directly, or chose Coin whose outcome was (5,5) or (8,2). To create this information condition, we listed (5,5), (8,2), and Coin on the proposal form, but instructed all participants that Proposers could only choose Coin by leaving all options unmarked. After Proposers made their choices, the experimenter in the room of Responders privately flipped a coin. On any forms on which the Proposer chose Coin, the experimenter marked (5,5) or (8,2), based on the coin flip outcome. Thus, all participants understood that the Responder would be unable to distinguish forms on which the Proposer chose (5,5) or (8,2) directly from forms on which the Proposer chose coin.

We followed the same fixed order as in [5] as 1) full, 2) private, and 3) limited because a different ordering led to confusion in pilot studies with adults. Given that children constitute our sample, we wanted to minimize the risk of such confusion. Furthermore, because we did not provide Proposers with feedback between conditions, and because participants only played three games, we expected any effects of learning without feedback [24] to be minimal. In other words, Proposers never knew the Responders' decision about their offer.

### False belief task

We administered a modified version of the second-order false belief task called “Look Prediction”, which also includes the evaluation of first-order false belief understanding [25], [26]. The child is told a story (with drawings) about Maria and Gianni who are playing with a toy. Maria puts the toy in a wardrobe and leaves the room, and while she is away Gianni changes the location of the toy, putting it under the bed. The story is stopped and the child is asked where Maria will look for the toy once back in the room (first order false belief question). The child is then asked to justify the response, and is further given memory and reality control questions to assess understanding of the story. Then, the story is resumed, with Maria returning to the room. From the open door she sees Gianni as he is moving the toy under the bed, though Gianni does not see Maria. The child is then asked where Gianni thinks Maria will look for the toy once back in the room (second order false belief question). Again, the child must justify this answer, and is asked memory and reality control questions.

The sample was partitioned according to the answers to the control questions. No child was excluded. Both for first order and second order false belief understanding, the question regarding false belief was scored 1 if correct and 0 if incorrect. The justification question was scored 0 if incorrect, 1 if correct and without reference to the mental activity, and 2 if correct and with reference to the mental activity. A total score was computed both for first order and second order false belief understanding (range 0-3). According to this range, children were grouped in Low (0–1) and High (2–3) second order false belief understanding.

### Fairness judgments and beliefs

We coded Responders' normative judgments about the fairness of each proposal by condition (fair or not fair). For each condition and proposal, we also coded whether each Responder and Proposer believed the majority of Responders said the proposal was fair (majority said was fair or majority said was not fair); that is, we recorded each child's beliefs about the distribution of the Responders' normative judgments.

### Design and Analysis

We employed a 3×2×2 design, crossing information (full, private, and limited, within-participants) with salience (non-salient and salient, between-participants) and age (8 or 10 years old). Primary dependent variables included the Proposer's choice of (5,5), (8,2), or Coin and the Responder's decision to accept or reject, which we respectively analyzed as multinomial or binomial responses. Nested model comparisons were based on the likelihood ratio test. We tested for a participant-level zero-mean random intercept to model the grouped nature of the data to account for potential within-participant correlation [27], and also separately estimated White-Huber robust standard errors. Finally, we separately analyzed the effects of several covariates by condition, including theory of mind performance (low or high), fairness beliefs by choice (0 = majority said the choice was not fair, 1 = majority said the choice was fair), age (in months), and gender.

## Hypotheses

Consistent with [5], we predicted that:

H1: Proposers will choose Coin more frequently in the Full information condition and less frequently than 5-5 and 8-2 both in the Private and in the Limited conditions, because in the last two conditions Proposers will take advantage of the informational opacity about, respectively, the existence and the use of Coin.

H2: Proposers will choose (8,2) more frequently in the Limited information condition than in the Full information condition. The (8,2) will be the most frequent choice in the Limited condition, because Proposers will know that Responders will not be able to figure out whether the 8,2 offer is intentional or results from the Coin flip.

H3: In the Salient condition, we expected: a) more 5-5, b) more Coin offers, and c) less 8-2 offers in any condition, because focusing on a norm of fairness should improve norm compliance.

Furthermore:

H4: older children who act as Proposers will behave more like adults, because strategic reasoning improves with age. More specifically, older children will comply with the norm depending on the fact that different information conditions entail different possibilities of discovering transgression (in the Full condition transgressions are immediately identified, whereas in the Limited condition they can be only suspected). Instead, younger children will comply with the norm more consistently in all the information conditions, because they have a less sophisticated strategic reasoning, and therefore they think that a potential transgression will be always discovered.

H5: A combined effect of age and second order false belief understanding (high-low) will affect the decision. Proposers with high second order false belief understanding will be more “Machiavellian”, i.e. they will try to take advantage of the asymmetric access to information of the partner in the Limited condition in order to disregard the fairness norm at no cost for themselves.

H6: Responders in the Full condition will refuse the (8,2) offer from the direct choice of the Proposer more frequently than the (8,2) offer deriving from Coin, because children progressively include intentions and expectations into the decision process. Furthermore, they will accept (8,2) offers more frequently in the Limited condition than in the Full condition because they will not be able to figure out whether the offer is intentional or random. Finally, we expect that these behaviors will depend on age and high level of second order false belief understanding, because these two factors will make children less consequentialist, i.e., more sensitive to intentions and disposed to believe that the (8,2) offer results from the Proposers' fair choice of Coin.

H 7: We expect that Responders will make a decision that is coherent with their personal normative judgment, similarly to the finding of Bicchieri and Chavez with adults [5].

## Results

### Proposer Behavior

Choice by Condition, Salience, and Age. Relative to constant choice proportions (the null hypothesis), there was a significant effect of condition on choices (χ2(4) = 9.74, p = .045), but not of salience (χ2(2) = 0.41, p = .82) or age (χ2(2) = 0.20 p = .91). Whereas adults were more inclined to choose (5,5) and less inclined to choose (8,2) across information conditions in the salience treatment (Bicchieri & Chavez, 2010), children's behavior was not influenced by salience. Moreover, there were no significant interactions of condition and salience (χ2(4) = 3.47, p = .48), condition and age (χ2(2) = 4.11, p = .13), salience and age (χ2(4) = 5.96, p = .20), or Condition×Salience×Age (χ2(4) = 3.10, p = .54).

Therefore, we estimated a multinomial logit model of choice on condition with a participant-level random effect and a separate multinomial logit model with robust standard errors, as shown in Table 1.

**Table 1:** Multinomial logit model of choice by condition.

(5,5)	Coefficient	ML SE	*p*	Robust SE	*p*	Random Effect SE	*p*
Intercept	−0,53	0,31	0,0804	0,23	0,0217	0,31	0,0804
Private	0,92	0,45	0,0430	0,36	0,0101	0,45	0,0430
Limited	0,29	0,29	0,4386	0,34	0,3919	0,44	0,5100
(8,2)							
Intercept	−1,76	0,48	0,0003	0,44	0,0001	0,48	0,0003
Private	1,69	0,61	0,0057	0,54	0,0018	0,61	0,0057
Limited	0,92	0,61	0,1325	0,55	0,0910	0,61	0,1325

Notes. The reference level for choice is coin, and for condition is full. Standard errors are shown for maximum likelihood (ML), White-Huber robust estimation, and a participant-level random intercept model. The participant-level random effect variance was not significant (σ_p_^2^ = 11^−12^, p = 1.0, n.s.). Coefficients were the same to two decimal points in all three models.

**Figure 1:**
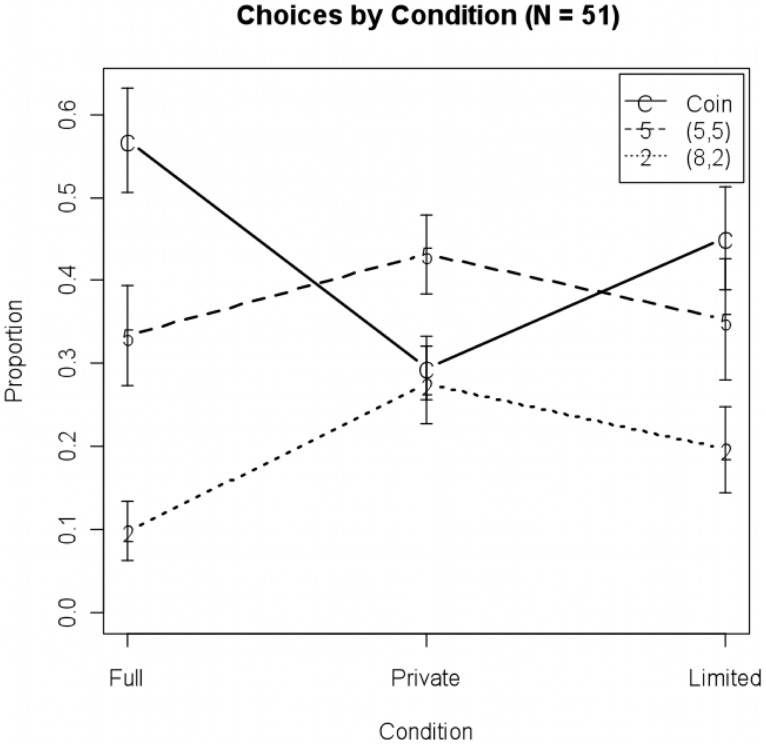
Choice proportions by condition. Error bars represent +/−1 bootstrap standard errors.

The random effect variance was not significant (σ_p_^2^ = 11^−12^, p = 1.0, n.s.), and robust standard error estimates were similar to classical standard error estimates, so we based inference on a plain multinomial logit model of choice on condition. Figure 1 shows choice proportions and error bars corresponding to the model of choices by condition (residual deviance = 309.93, df = 147, random effect variance σp2 = 11–12, p = 1.0, n.s.).

### Pairwise Comparisons Across Conditions

As is clear from Figure 1, (5,5) choices were stable across conditions. This is different from adults' results, where (5,5) in the limited and full conditions was chosen with less frequency than (8,2) and Coin, respectively. As hypothesized, the proportion of coin choices in the full information condition was significantly higher than in the private condition (.57 vs. .29, p = .003). However, we found no support for the hypothesis that coin choices were more frequent in the full vs. limited conditions (.57 vs. .45, p = .12). There was marginal support for the hypothesis that the proportion of (8,2) choices was higher in the limited vs. full conditions (.20 vs. .10, p = .09). Proposers were clearly sensitive to changes in information conditions and displayed strategic behavior in the Full and Private conditions. However, their behavior in the Limited condition was very different from the more Machiavellian adult behavior.

### False belief understanding

There was no effect of Proposer second order false belief understanding (FBU) grouping (high vs. low) on choice (χ2(2) = 1.21, p = 0.5458), nor was there a significant interaction between second order false belief understanding grouping and information condition (χ2(4) = 6.43, p = 0.1693). Also, a combined effect of age and second order false belief understanding grouping on decisions was not found (χ2(2) = 2.09, p = 0.3517).

### Fairness Judgments

Table 2 shows Responders' personal normative judgments (first order normative beliefs) about the fairness of each proposal by condition. In line with previous findings for adults [5], almost all Responders considered (5,5) to be fair across conditions. Between 6% and 10% of children considered (8,2) to be fair, whereas between 14% and 17% of adults found (8,2) to be fair. The most striking difference between the two populations was the children's nearly unanimous judgment of Coin as fair compared to lesser agreement over the fairness of coin in adults (94% vs. 57% judge coin as fair in the limited condition, χ2(1) = 14.2, p = .0002, and 96% vs. 64% judge coin as fair in the full condition, χ2(1) = 16.1, p<.0001). Fairness beliefs did not depend on age, gender, or false belief understanding. Clearly, children were uniform in thinking that both (5,5) and Coin are fair choices. In fact, when we look at the answers that Proposers gave after they made their choices, the two most frequent justifications of those choosing Coin were: 1. *It is okay because it gives both of us an equal chance*, and 2. *I chose it because I was undecided, I did not know what to do*. In both cases, the choice of Coin was perceived as fair, even by those that openly admitted they wanted to avoid the responsibility of choosing one of the two other options.

**Table 2:** Personal normative beliefs (fairness judgments) of Responders.

	Choice					
Condition	(5,5)		(8,2)		Coin	
Full	98.0%	50/51	5.9%	3/51	96.1%	49/51
Private	96.1%	49/51	7.8%	4/51		
Limited	94.1%	48/51	9.8%	5/51	94.1%	48/51

Each cell contains the proportion (fraction) of Responders who indicated that the choice was fair. Note. Each cell contains the proportion (fraction) of Responders who indicated that the choice was fair.

### Second-order Beliefs (Normative expectations) about Responders' fairness Judgments

**Figure 2:**
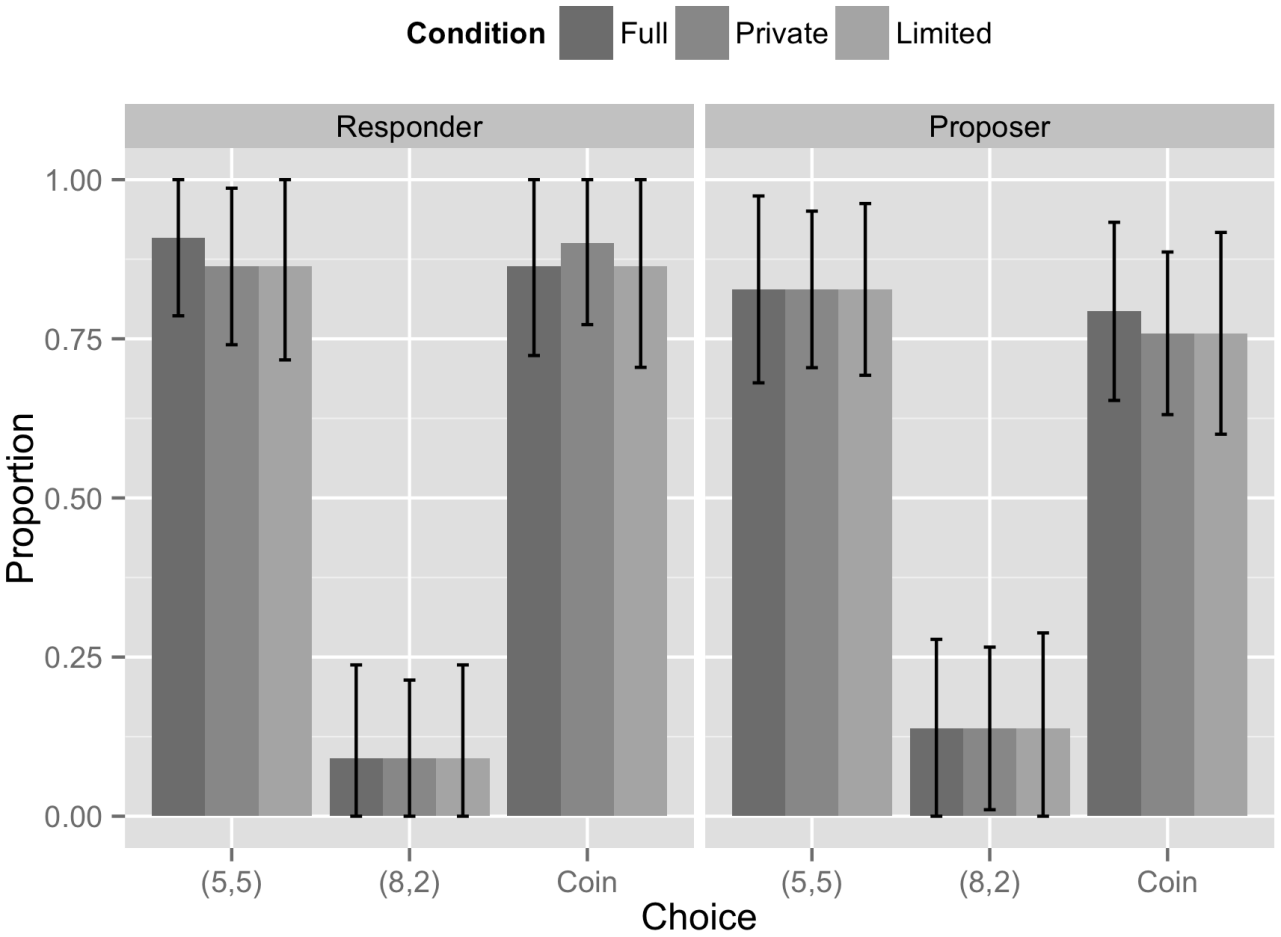
The proportion of Responders (left graph) and Proposers (right graph) who believed the majority of Responders indicated each choice was fair, by condition. Error bars are unadjusted 95% confidence intervals.

Figure 2 shows the distribution of Proposers' and Responders' beliefs about Responders' personal fairness judgments (see Table 2) by condition, for the salient treatment only (as these variables were recorded for Proposers only in the salient condition). There is a remarkable degree of agreement across Responders' and Proposers' second-order normative beliefs. Averaging over conditions, 87.9% of Responders and 82.8% of Proposers believed the majority of Responders indicated (5,5) was fair. 9.1% of Responders and 13.8% of Proposers believed the majority of Responders indicated (8,2) was fair; 87.6% of Responders and 77% of Proposers believed the majority of Responders indicated Coin was fair. Like the adults in [5], children show a remarkable consistency in second-order normative beliefs. Since consistency in normative expectations (second-order beliefs) strongly suggests that a social norm is in place, we may conclude that children think that a norm of fairness includes both equal division and a fair random procedure (tossing a fair coin, in our case). Again, there was no significant difference in the beliefs of low and high false belief understanding (FBU) subjects, suggesting that awareness of fairness norms may not depend upon having a false belief comprehension of high or low sophistication.

### Responder Behavior

Logistic regressions of rejections on offer, condition, Responder false belief understanding (FBU) grouping, and their two- and three-way interactions revealed a main effect of offer (χ^2^ (2) = 83.05, p<.0001), but no effects of condition (χ^2^ (2) = 0.63, p = .7317), FBU grouping (χ^2^ (1) = 1.25, p = .2629), Offer x Condition (χ^2^ (2) = 2.32, p = .3139), Offer x FBU Group (χ^2^ (1) = 2.45, p = .1179), Condition x FBU Group (χ^2^ (2) = 0.31, p = .8587), or Offer x Condition x FBU Group (χ^2^ (2) = 1.06, p = .5889). Table 3 tabulates Responder rejections by offer and condition.

**Table 3:** Rejection rates and frequencies by offer source, offer, and condition.

	Offer			
Offer source	(5,5)		(8,2)	
Full:				
Direct choice	0.0%	(0/17)	80.0%	(4/5)
Coin flip	16.7%	(2/12)	76.5%	(13/17)
Private	6.9%	(2/29)	86.4%	(19/22)
Limited	16.7%	(5/30)	76.2%	(16/21)

Responders uniformly rejected offers of (8,2) across all conditions, irrespective of the reason why that outcome obtains, as reported in Table 3. For example, in the Full information condition, Responders who received a low payoff from a coin toss knew it was the result of a random event. They knew the outcome was not the result of an intentionally mean choice, but just bad luck. Though almost all Responders uniformly considered Coin a fair choice, this judgment was not mirrored in the rejection rates. Adults, on the contrary, are much more consistent, as they accept a low payoff when it is the result of a random choice [5]. Adults, in other words, are well aware of intentions and causal processes. For example, only 18.2% of adults rejected (8,2) in the Limited Condition, suggesting that they entertained the possibility that (8,2) was a result of chance. Children however completely discount this possibility and reject (8,2) in the same high proportion (76.2% and 76.5%) in the Limited and Full Conditions, even when (8,2) is known to be the result of a coin flip.

It looks as if kids have acquired the sense that norms of fairness may mean different things, but do not use this knowledge to evaluate outcomes. When they judge outcomes, they act as pure consequentialists. As further support to this interpretation, we found that in the Full condition all the children who rejected 8,2 from Coin justified their choice by making a clear reference to inequity aversion, such as *I want the same number of tokens* or *it is not fair that he/she has more tokens than me*. Our result is in line with other data showing that when an unfair offer is the result of an unintentional event, such as a roulette outcome, it is rejected by children as much as if it results from an intentional choice [21].

## Conclusions

Part of the first hypothesis, that Coin would be chosen more frequently in the Full information condition, was supported by the data. However, we also expected that Coin would be chosen less frequently than 5,5 and 8,2 both in the Private and in the Limited conditions. Coin was indeed chosen much less than 5,5 in the Private condition, but it was the most frequent choice in the Limited condition. Moreover, Coin and 8,2 were chosen with the same (low) frequency in the Private condition.

The second hypothesis was only partially supported by the data: (8,2) choices were not more frequent than other choices in the Limited condition. Yet the data show a tendency of (8,2) to be more frequent in the Limited condition than in the Full condition.

The remaining hypotheses were not supported by the data.

As for the Proposer perspective, children's behavior shows awareness of fairness norms, and the capability to use them in a self-serving way. Whereas Coin is the most frequent choice in the Full condition, it precipitously declines in the Private condition, because children are aware that Responders do not know that Coin is available in that condition. Proposers know that an offer of (8,2) in the Private condition would be interpreted as intentional, and thus is likely to be rejected. In the Full condition, Proposers exploit their belief that a large majority of Responders believes Coin is fair. Yet if we look at the expected utility of Coin, it is quite clear that it is less fair than (5,5) for Responders. Also, the fact that Coin is the most frequent choice in the Limited condition (where it is public knowledge that Coin is available) suggests that children are not as Machiavellian as adults, probably because the Limited information context is particularly difficult to grasp. In order to choose (8,2) in Limited, a child must not only be aware that the Responder knows that Coin is available, but must also believe that the Responder believes that Coin is fair and will accept a payoff of 2 as the result of chance.

The complexity of mental representations that support the exploitative (8,2) choice in Limited may be beyond recursive second-order thought. It may also be the case that such complexity creates an ‘overload’, in the sense that even if the child possesses an adequate theory of mind, it is not sufficiently robust to allow the child to manage all the multifaceted representational content at the same time. This might explain why the second-order false belief understanding (high or low) has no impact on strategic decision making in our experiment. The Private and Full conditions, on the other hand, are quite simple to grasp, so that first order false belief understanding – sufficiently mastered in the age groups considered – is enough to manage such conditions.

With regard to the Responder perspective, children reject the (8,2) offer irrespectively of its origin and information condition: they seem to ignore the role of intentionality vs. chance, as well as the presence of different levels of informational transparency. This may be due to a difficulty in coordinating all these elements (intentionality and information manipulations) in the decision process, which would lead children to adopt a simpler consequentialist approach. This difficulty could explain the incoherence between personal normative judgments and behavior: children think that the option of tossing a coin to determine an offer is completely fair but if the result of such procedure is unfavorable, they reject it. Adults, as found by Biccheri & Chavez [1], are more consistent, as they rarely refuse an unfair outcome resulting from a coin toss.

A first possible explanation of our result refers to the ‘outcome bias’ effect, which is common also in adult judgments. For example, Cubitt et al. [28] show that, in judging the outcomes of social dilemmas, people who express a negative judgment of free riding give greater weight to the consequences of actions rather than the intentions behind those actions. In line with our findings, Gino et al. [29] found that adults, acting as third-party observers, judge an unfair offer of (9,1) as more *morally* unacceptable than a fair offer of (5,5), even if both offers are the result of a coin toss in a Dictator Game. Adults, however, tend to accept the unfair results of a fair procedure, even if those who accept the unfair offer show significant neural activations in the insula, a brain structure devoted to processing the negative emotion of disgust [30]. This evidence suggests that adults experience a conflict as well, because although they ‘rationally’ accept an unfair offer from a non-human partner they still have a negative emotional reaction. Children, however, do not seem able to override the negative emotion elicited by the unfairness of the offer resulting from the coin toss, as such emotion is stronger than their belief that Coin is fair [21]. This conflict is evident in the Responders' rejection, but not in the Proposers' choices. This difference is not surprising, since Proposers do not experience a conflict between emotion and belief, and can thus assign full weight to the judgment that Coin is fair, and perceived as fair by all the parties.

In children, the separation of desire and belief may not yet be complete, and thus the conflict is ‘played out’ in rejecting an offer that, though stemming from a fair procedure, is perceived as unfair. In fact, the development literature has consistently shown that desire plays an important role driving children's beliefs and behaviors during the first years of development. Desire often overrides belief, creating problems in theory of mind reasoning: children fail to solve the classical false belief task because the attribution to the story character of a desire for the hidden object determines a distortion in the attribution of false belief. In our study the desire for an equal outcome overrides the belief about fair procedures, and severs the connection between beliefs and behavior.

The inconsistency between fairness beliefs and behaviors in our children is in line with a study of children aged between 3 and 8 that found that children endorse fairness norms related to sharing, but act inconsistently with these norms [31]. Again, this result is evidence of strong inequity aversion, and suggests that inequity aversion evolves much earlier than our capability of making finer distinctions of procedural fairness and applying them to the evaluation of outcomes. Children may thus learn fairness norms *before* they become able to accept their consequences and make adults' distinctions about the acceptability of outcomes depending upon how they originated. The split between mutual normative expectations and Responders' behavior suggests a split between an early response to outcomes and a much later ability to evaluate outcomes according to a culture's norms that have been learned quite early, but not fully absorbed.

A second possible explanation for the discrepancy between fairness beliefs and decision in children may refer to the fact that the fairness beliefs may result from an evaluation of the coin toss as fair “in isolation”, whereas the decision to refuse the 8,2 offer deriving from coin may depend on the comparison of such offer with the other options, i.e. children might think that the Proposer would have been much more fair by offering 5,5 directly. If it cannot be excluded that children might have expressed the fairness judgment evaluating the coin toss procedure *per se*, it is also true that the absence of a correlation between the Responder's behavior and second order false belief reasoning makes the consequentialist interpretation the most plausible.

Overall, our findings provide useful cues to better understand children's decision-making behavior not only in the lab, but also in real life. In fact, even if some evidence exists showing that self-selected students are an appropriate subject pool for the study of social behavior in bargaining games [32], our sample of children is not affected by the problem of self-selection, since they are not volunteers. However, our findings are subject to some limitations. First, in order to avoid learning effects, the actual procedure did not provide Proposers with feedback between conditions. The modification of this methodological option would allow exploring the disposition of Proposers to take advantage of Coin in Full but not in Private when we provide feedback about the Responder's decision. We also presented the information conditions in a fixed order (Full, Private, and Limited) because we wanted to replicate Bicchieri and Chavez [5] in order to compare children to adults. Future research should address the possible influence of order on normative beliefs by means of a different paradigm. Second, to distinguish between an understanding of coin toss as a fair procedure or as a fair offer the questionnaire about fairness beliefs should be formulated in a comparative way, i.e. asking for a fairness judgment of each offer option compared to the other available options. Third, with respect to the theory of mind, we did not find a significant effect of second order false belief understanding on the norm of fairness. Although our evidence may suggest that theory of mind – operationalized as false belief understanding – does not much influence these decision processes, it is possible that the task employed does not grasp the full complexity of meta-representational ability. Future research should use a larger battery of tests of theory of mind, also including qualitative measures of the ability to adopt the perspective of the other person and to empathize, which are involved in social bargaining in adults [33].

Concluding, children show a developing awareness of the fact that norms about fairness and normative expectations contribute to decision-making behavior. Furthermore, children embody the popular quote “easier said than done”, as they reject the unfair outcome of a decision judged as fair. They know what is fair, but they do not know how to act consistently with their judgment.

## Supporting Information

Document S1 **Ultimatum game instructions and familiarization.**
(DOCX)

Document S2 **Extended description of method and procedure.**
(DOCX)

Document S3 **Questionnaire about Responders' first-order normative beliefs.**
(DOCX)

Document S4 **Questionnaires about Proposers' and Responders' second-order normative beliefs (expectations).**
(DOCX)
