# Supplementary material for: Fairness Norms and Theory of Mind in an Ultimatum Game: Judgments, Offers, and Decisions in School-Aged Children
Source: PLoS One. 2014 Aug 13;9(8):e105024. doi: 10.1371/journal.pone.0105024 (PMC4132049; doi:10.1371/journal.pone.0105024)
Supplement: Document S1 — Ultimatum game instructions and familiarization. (DOCX) [file pone.0105024.s001.docx]

**Document S1 - Ultimatum game instructions and familiarization**

Materials:

- 10 tokes (same shape, colour, size).
- Candies and stickers.

Experimenter (E.): We are going to play a game. Please, tell me what do you prefer: these candies or these stickers? (candies and stickers visible on the table).

Child (C.): I prefer candies.

E.: Ok. In this game you can win tokens (10 tokens visible on the table), that at the end of the game can be converted into candies for you. Listen: two tokens are equal to one candies (E. makes the example using two tokens and one candy). Is that clear?

C.: Yes.

E.: Ok. Now I am going to explain you the game. In this game there are two players: one is called the Proposer, i.e. the child who decides how to divide the 10 tokens. The other one is called the Receiver, i.e. the child who decides whether to accept or to reject the offer. Be careful to this important rule: if the Receiver accepts the offer, the tokens are divided as proposed, so each child winds some tokens; if the Receiver rejects, nobody gets anything. Is that clear? Ok, let’s play some trial games before doing the real ones.

So, here are the 10 tokens. I am the Proposer and you are the Receiver. My proposal is: 5 tokens for you (the E. gives 5 tokens to the child) and 5 tokens for me (the E. keeps 5 tokens).

What do you decide? Do you accept or do you refuse?

If the child accepts, E. says: “Ok, you accepted. So you get 5 tokens and I get 5 tokens”.

If the child rejects, E. says “Ok, you refused. So nobody gets anything (the tokens are put away)”.

The E. continues: “Ok, let’s play another trail. Now my proposal is: 2 tokens for you (the E. gives 2 tokens to the child) and 8 tokens for me (the E. keeps 8 tokens).

What do you decide? Do you accept or do you refuse?

If the child accepts, E. says: “Ok, you accepted. So you get 2 tokens and I get 8 tokens”.

If the child rejects, E. says “Ok, you refused. So nobody gets anything (the tokens are put away)”.

*ATTENTION: the order of the two trials (5-5 and 8-2) was counterbalanced across subjects.*

Now, let’s change: you are the Proposer and I am the Receiver. Here are the 10 tokens. What is your proposal?

*The child makes his/her offer, and the E. always accepts it, making the division of the tokens always clear on the table.*

*Familiarization with coin toss*

E.: Ok, now I am going to tell you one last thing. Sometimes, the Proposer (i.e. the person who makes the offer) can flip a coin to make the offer:

- if the result is head, the offer is 5-5;
- if the results is tail, the offer is 8 (for the Proposer) -2 (for the Responder).

The E. makes some trials with the coin to show how it works.

*Disclosure*

E.: So, is everything clear? If you have some doubts please do not hesitate to ask me, because it is important that all the rules are clear before playing this game.

Ok, let’s start with the game.

You are extracted to play in the role of Proposer/Receiver. This means that in all the game rounds you will always play as Proposer/Receiver.

*If the child is extracted to play as Proposer, the E. says:*

So, you will play as Proposer. You are free to choose the division that you prefer (among 5-5, 8-2, coin toss), nobody (neither the other child that will receive your offer, nor me, your teacher, your parents, your friends…) will be able to know which offer you did.

How? Look: here we have some tickets with symbols: one has a star, one has a cross and so on. You pick one up, look at the symbol and write it on the back of your proposal form. In this way, nobody will ever know that it was you (name of the child) who made a certain offer.

Ok?

Good, here we go!
