# Supplementary material for: Fairness Norms and Theory of Mind in an Ultimatum Game: Judgments, Offers, and Decisions in School-Aged Children
Source: PLoS One. 2014 Aug 13;9(8):e105024. doi: 10.1371/journal.pone.0105024 (PMC4132049; doi:10.1371/journal.pone.0105024)
Supplement: Document S2 — Extended description of method and procedure. (DOCX) [file pone.0105024.s002.docx]

**Document S2-Extended description of method and procedure**

The Ultimatum game was played with random assignments of Proposers and Responders.

All participants played three modified Ultimatum games, each with a different partner (no feedback between conditions). For each of the three rounds tokens were converted into candies or stickers to each pair, according the child’s preference.

Proposal form:

5-5: 5 tokens for Proposer, 5 tokens for Responder

8-2: 8 tokens for Proposer, 2 tokens for Responder

Coin toss: a fair coin toss is used to determine whether 5-5 or 8-2 is offered

**Game 1: Full/Public Information**

- All participants knew about the coin toss.
- Responders saw the coin toss outcome.

**Game 2: Private Information**

- Only Proposers knew about the coin toss option and outcome.

**Game 3: Limited Information**

- All participants knew about the coin toss option.
- No participants saw the coin toss outcome.

Before playing the three Ultimatum games, each child was explained the rules of the game and was involved in a trial test, to be sure that he/she had fully understood the procedure.
