# Supplementary material for: Fairness Norms and Theory of Mind in an Ultimatum Game: Judgments, Offers, and Decisions in School-Aged Children
Source: PLoS One. 2014 Aug 13;9(8):e105024. doi: 10.1371/journal.pone.0105024 (PMC4132049; doi:10.1371/journal.pone.0105024)
Supplement: Document S3 — Questionnaire about Responders' first-order normative beliefs. (DOCX) [file pone.0105024.s003.docx]

**Document S3 – Questionnaire about Responders’ first-order normative beliefs**

There are 30 children who play as Proposers, i.e. who make the offer.

Guess how many of them will choose:

1. 5 tokens for the Responder and 5 tokens for themselves: ……….
2. 2 tokens for the Responder and 5 tokens for themselves: ………..
3. Tossing a coin (remember: head, 5-5; tail, 8-2): ………………...

Now, for each offer, tell me if it is fair or it is unfair. You can say that only one offer is fair, or that two offers are fair, or that all three offer are fair, or that none is fair. Feel free to judge, your answer will not make you earn or loose any tokens.

1. 5 tokens for you and 5 tokens for the Proposer: **yes, it is fair no, it is unfair**
2. 2 tokens for you and 8 tokens for the Proposer: **yes, it is fair no, it is unfair**
3. Tossing a coin (remember: head, 5-5; tail, 8-2): **yes, it is fair no, it is unfair**
