# Supplementary material for: Fairness Norms and Theory of Mind in an Ultimatum Game: Judgments, Offers, and Decisions in School-Aged Children
Source: PLoS One. 2014 Aug 13;9(8):e105024. doi: 10.1371/journal.pone.0105024 (PMC4132049; doi:10.1371/journal.pone.0105024)
Supplement: Document S4 — Questionnaires about Proposers' and Responders' second-order normative beliefs (expectations). (DOCX) [file pone.0105024.s004.docx]

**Document S4 – Questionnaires about Proposers’ and Responders’ second-order normative beliefs (expectations)**

Before each game half of the Proposers and half of the Responders received an incentive-based questionnaire that measured their second-order normative beliefs (expectations). Because the questionnaire placed focus on fairness norms, we labeled this condition the *salient condition*.

*Version for the Proposers*

Now I am going to ask you some information. Be careful, because you will earn an extra-token for each correct answer you will give me.

Each child who receives the offer is asked to say if the offer “5-5” (i.e. 5 tokens for him/her and 5 tokens for the proposer) is fair.

According to you, on 30 children who receive the offer, how many of them will say that “5-5” is fair?

- all of them, i.e. 30
- more than half of them, i.e. more than 15
- less than half of them, i.e. less than 15
- nobody

Each child who receives the offer is asked to say if the offer “8-2” (i.e. 2 tokens for him/her and 8 tokens for the proposer) is fair.

According to you, on 30 children who receive the offer, how many of them will say that “8-2” is fair?

- all of them, i.e. 30
- more than half of them, i.e. more than 15
- less than half of them, i.e. less than 15
- nobody

Each child who receives the offer is asked to say if tossing a coin to have the offer is fair.

According to you, on 30 children who receive the offer, how many of them will say that “tossing a coin to have the offer” is fair?

- all of them, i.e. 30
- more than half of them, i.e. more than 15
- less than half of them, i.e. less than 15
- nobody

*Version for the Responders*

Now I am going to ask you some information. Be careful, because you will earn an extra-token for each correct answer you will give me.

The are 30 children who receive the offer from another child, just like you (i.e. they play as Responders). Guess how many of them will say that each of the following offers is fair:

1. 5 tokens for him/her and 5 tokens for the other child (the Proposer).

They will say that it is fair:

- all of them, i.e. 30
- more than half of them, i.e. more than 15
- less than half of them, i.e. less than 15
- nobody

1. 2 tokens for him/her and 8 tokens for the other child (the Proposer).

They will say that it is fair:

- all of them, i.e. 30
- more than half of them, i.e. more than 15
- less than half of them, i.e. less than 15
- nobody

1. The offer comes out from tossing a coin: head, each child gets 5 tokens; tail, the proposer gets 8 and the other child gets 2.

They will say that it is fair:

- all of them, i.e. 30
- more than half of them, i.e. more than 15
- less than half of them, i.e. less than 15
- nobody
